# Metabolic syndrome and associated factors among severely ill psychiatric and non-psychiatric patients: a comparative cross-sectional study in Eastern Ethiopia

**DOI:** 10.1186/s13098-021-00750-4

**Published:** 2021-11-10

**Authors:** Dilnessa Fentie, Tariku Derese, Bekele Yazie, Yibeltal Getachew

**Affiliations:** 1 School of Medicine, College of Medicine and Health Sciences, Dire Dawa University, P.O. Box 1362, Dire Dawa, Ethiopia; 2 Department of Public Health, College of Medicine and Health Sciences, Dire Dawa University, Dire Dawa, Ethiopia; 3 Department of Psychiatry, College of Medicine and Health Sciences, Dire Dawa University, Dire Dawa, Ethiopia

**Keywords:** Metabolic syndrome, Dilchora Hospital, Eastern Ethiopia, Psychiatric disorders

## Abstract

**Background:**

Metabolic syndrome is a major public health challenge in both developed and developing countries. The burden of this disease is high, even in patients with psychiatric disorders. However, very little is known about the association between metabolic syndrome and psychiatric illness in Ethiopia. Therefore, the aim of this study was to investigate the magnitude of metabolic syndrome and its components among psychiatric clients.

**Methods:**

A comparative cross-sectional study was undertaken between psychiatric patients and age—and sex-matched non-psychiatric controls at the Dilchora referral hospital. The study included 192 study participants (96 psychiatric patients and 96 non- psychiatric controls from general medical and surgical patients). The National Cholesterol Education Program: Adult Treatment Panel III criteria were used to diagnose metabolic syndromes. The data were cleaned and analyzed using the Statistical Package for Social Sciences, Version 21. All intergroup comparisons for continuous data were performed using an independent sample t-test, whereas categorical data were analyzed using the Chi-square test. Logistic regression analysis was used to identify the association between metabolic syndrome and the associated variables.

**Results:**

The magnitude of metabolic syndrome among psychiatric patients was 36.5% (95%CI: 27.6, 47.4) compared to non-psychiatric control patients, 21.9% (95%CI: 13.5, 30.3)**, **p = 0.02. The prevalence of MetS components, such as waist circumference (25.0% vs. 14.3%), lower-high density lipoprotein level (35.4% vs. 20.8%), higher systolic blood pressure (41.7% vs. 29.2%) and higher fasting blood glucose (40.6% vs. 18.8%) showed statistically significant differences between the exposed and non-exposed groups. Age greater than 50 years (AOR: 2.8, CI: 1.14, 20.0, p < 0.05); being female (AOR: 7.4, CI: 2.0, 27.6, p < 0.05), being urban residence (AOR: 6.4, CI: 2.2, 20.6, p < 0.05), ever alcohol intake (AOR: 5.3, CI: 1.3, 21.2), being physically inactive (AOR: 3.52, CI: 1.1, 12.9, p < 0.05) and family history of hypertension (AOR: 2.52, CI: 1.1, 12.2, p < 0.05) were independent predictors of metabolic syndrome (p < 0.05).

**Conclusions:**

There is a high burden of metabolic syndrome and its components in patients with severe psychiatric disorders. Therefore, screening and mitigation strategies for metabolic syndrome and their components should be implemented in the management of psychiatric disorders.

## Plain language summary

Metabolic syndrome is a risk factor for cardiovascular disease that has public health issues, which places social, economic, and disease conditions in the community within the geographical region of sub-Saharan Africa, including Ethiopia. Patients with psychiatric illness have a 2–threefold risk of morbidity and mortality from metabolic syndrome compared to the general population. Here, we look at range of the possible reasons psychiatric patients have less physical activity due to functional disorders or psychotropic medication, psychological stress, excessive alcohol intake, and inadequate medical care. We assessed the burden of metabolic syndrome and its components among psychiatric patients as compared to non-psychiatric individuals in Eastern Ethiopia for a period of 6 months. There has been an alarming increase in the burden of metabolic syndrome and its components among patients with psychiatric illness. Hence, our results allow readers to aware of the burden of metabolic syndrome and factors involved in the development of the syndrome among psychiatric clients.

## Introduction

Central obesity, hypertension, type 2 diabetes mellitus (T2DM), and atherogenic dyslipidemia are among the cardiometabolic risk factors that make up metabolic syndrome, often known as insulin resistance syndrome [1, 2].Central obesity, leads to an increase in the flow of free fatty acids and cytokine proinflammatory mediators that contribute to the development of insulin resistance [3, 4]. The inflammatory cytokines interleukin-6 and tumor necrosis factor alpha released from expanded visceral adipose tissue impair insulin signaling due to increased phosphorylation of insulin receptor substrate-1 (IRS-1) [5, 6].

Insulin resistance, which is a key underlying feature of type 2 diabetes mellitus, has been shown to be associated with dyslipidemia (hypertriglyceridemia and hypo-high-density lipoprotein cholesterol (HDLc)) and systemic hypertension [7]. Dyslipidemia is defined as raised triglycerides and decreased high- density lipoprotein cholesterol levels. HDL receives triglycerides from very low- density lipoprotein with the activity of the cholesterol ester transport protein, HDL particles are rich in triglycerides, which can be rapidly cleared by hepatic lipase. Insulin resistance reduces the retention of free fatty acids by adipocytes due to the lack of inhibition of insulin by hormone-sensitive lipase, and high rates of triglyceride synthesis [8, 9].

Metabolic syndrome is a major public health problem that has been recognized as a global epidemic by the World Health Organization [10, 11]. Metabolic diseases impact roughly 20%–25% of the world's population and are linked to a twofold increase in the risk of death and a threefold increase in the risk of heart attack [12, 13] and its prevalence is 2–3 times higher in people with psychiatric disorders [14–18].

Metabolic syndrome is a major public health challenge in both developed and developing countries due to rapid urbanization, an increasing aging population, sedentary lifestyle, and dietary changes that act together as a web of risk factors which entangles people in it and leads to several non-communicable diseases among psychiatrically ill patients.

In Ethiopia, the burden of MetS has not yet been estimated, especially for patients with psychiatric disorders. Therefore, the aim of our study was to determine the prevalence, and factors associated with MetS among people with psychiatric illnesses attending Dilchora hospital, in Eastern Ethiopia.

## Materials and methods

### Study setting and design

An institution-based comparative cross-sectional study was conducted among patients with psychiatric disorders and non-psychiatric individuals. The study was conducted at the Dilchora Hospital (DCH) from January 5 to June 10, 2021. Dilchora hospital is located 515 km away from the capital city, Addis Ababa, Ethiopia. The hospital's psychiatry unit provides service for more than 4500 clients per year, and 1250 clients have registered for a follow-up visit. Monthly patient flow in the clinic ranges from to 390–420 and weekly patient flow is estimated to be 110–160.

### Population of the study

Cases/Severe psychiatric disorders/exposed/-established diagnoses of a common psychiatric disorder include schizophrenia, schizoaffective disorder, major depressive disorder, and bipolar disorder [19].

Controls/Non-psychiatric study groups/non-exposed group/were age-and sex-matched individuals, who did not have any psychiatric diagnoses, and who attended outpatient departments for general medical or surgical treatment other than psychiatric disorders, or who had no lifetime diagnosis or treatment for a mental illness.

### Source population

For cases: All clients attending the DCH, psychiatric clinic for treatment or advice during the study period.

For controls: All clients attending DCH, medical or surgical out patient department for treatment or medical advice during the study period**.**

### Study population

For cases: All selected clients attending the DCH, psychiatric clinic for treatment or advice during the study period.

For controls: All selected clients attending DCH, medical or surgical out patient department for treatment or medical advice during the study period.

### Selection criteria

Both men and women, aged 18 years and older were included in the study. However, both study groups with a history of pregnancy, previously known heart failure, renal failure, hormonal contraceptive use, and HIV seropositivity were excluded from the study.

### Sample size determination

The sample size was calculated using the formula for a hypothesis test for analytical studies [20]. To calculate the sample size, we considered the overall prevalence of metabolic syndrome among psychiatric patients to be 28.9% [21] and the prevalence of metabolic disorders among non-psychiatric populations to be 12.5% [22]. The estimated sample size for two-sample comparison of proportion at 80% power and 95% confidence level of certainty, and assuming a 10% non-response rate, the required sample size was 192 study participants (96 psychiatric patients and 96 non-psychiatric controls).

$$ {\text{n }}\left( {\text{each group}} \right) \, = \, \frac{{\left( {{\text{ r}} + {1}} \right) {\kern 1pt} \times {\kern 1pt} {\text{p}}* \times \left( {{1} - {\text{p}}*} \right)}}{{{\text{r }}\left( {{\text{p1}} - {\text{P2}}} \right)^{{2}} }} \times \, ({\text{Z}}\alpha + {\text{Z}}\beta )^{{{2} }} = {87} $$

Adding 10% of 87 = 9 + 87 = 96, n = Minimum sample size for each group, Z a/2 = critical value at 95% confidence level of certainty (1.96) (a constant), Zβ: This depends on power, (the probability of rejecting the null hypothesis when in fact false) for 80% this is 0.84, P1 = Proportions of MetS patients with psychiatric disorder, 28.9% = 0.289, P2 = Proportion of Mets among non-psychiatric patients, 12.5% = 0.125.

P* = average percentage between the two groups =  (p1 + p2)/2 = 42.3%/2 = 0.211, q = 1 − p* = 1–0.211 = 0.79, d = clinically meaningful difference between the two groups (p1−p2) = 0.289–0.125 = 0.174, r = ratio of diseased to non-diseased = 1, equal number of cases to controls.

### Sampling technique

Exposed study participants were selected consecutively to recruit all 96 severely ill psychiatric patients (diagnosed by psychiatrists or psychiatric clinical officers). After the data were collected from a Single case category, one corresponding age and sex matched non psychiatric controls were selected by convenience sampling.

### Operational definitions

Metabolic syndrome (MetS): is defined according to the modified United States National Cholesterol Education Program, Adult Treatment Panel (NCEP-ATP) III Guideline. Patients had at least three of the following risk features to be categorized under the MetS group: elevated waist circumference (WC) greater than or equal to 102 cm in men and 88 cm in women, elevated triglycerides (TG) greater than or equal to 150 mg/dl, reduced high-density lipoprotein-cholesterol, (HDL-C) less than 40 mg/dl in men and 50 mg/dl in women, elevated blood pressure greater than or equal to 130 mmHg systolic blood pressure (SBP) or 85 mmHg diastolic blood pressure (DBP), elevated fasting blood glucose (FBG) greater than or equal to 110 mg/dl, and metabolic disorder was diagnosed when ≥ 3 risk factors were present [23].

A vigorous-intensity activity: which causes a large increase in breathing or heart rate, if continued for at least 75 min (e.g., running, carrying, or lifting heavy loads, digging, or construction work) per week or engaging in leisure- time exercise at least three times a week for 30 min.

Moderate-intensity activity: which causes a small increase in breathing or heart rate, if continued for at least 150 min (brisk walking or carrying light loads) per week or walking for at least 30 min per day [24].Adults should perform at least 150 min of moderate-intensity physical activity or 75 min of vigorous-intensity physical activity per week, according to the WHO recommendation [25].

Smoking status: The study participants were all smokers (current, current daily, and past smokers), coded as (1 = yes) and non-smoker, coded as (2 = no).

Alcohol consumption: The study groups were ever consumers of any alcohol coded as (1 = yes) and lifetime abstainer coded as (2 = no). The ever- consumer/ consumer of any alcohol represents current alcoholic drinkers (past 30 days) which is coded as (2 = no and 1 = yes) and past alcoholic drinkers (drank in the past 12 months) coded as (2 = no and 1 = Yes).

### Data collection method and quality controls

All study groups were interviewed and blood tests were performed to determine sociodemographic, behavioral and clinical variables of the study groups. The questionnaires were adapted from the WHO STEP wise approach recommended for non-communicable disease surveillance. The data were collected by trained psychiatric nurses and laboratory technologists with close supervision of the investigators. The data collectors applied the same procedures to both psychiatric and non-psychiatric clients to collect data.

### Biochemical analysis

About 5–6 ml of the venous blood sample was collected after an overnight fast (8–12 h) and dispensed into fluoride oxalate tubes and vacutainer plain tubes for separation into plasma and serum, respectively. After clot formation, the blood specimen was centrifuged at 2000 revolution per minute (rpm) for 10 min within 30 min of collection and stored at 2–8 °C. Finally, and the serum was separated from the whole blood. Then, the serum was analyzed using an automated clinical chemistry analyzer for total cholesterol, high- density lipoprotein cholesterol, triglycerides and fasting blood sugar.

### Data quality control

Pretest of the data collection tool (questionnaire) was done by pre-testing 5% of questionnaires at the Goro health center, and necessary corrections were made prior to the actual data collection period. Trained psychiatric nurses and laboratory technologists participated in data collection, blood sample collection, and laboratory diagnosis. Blood samples were collected from all subjects between 8:00 and 12:00 h (a.m.) after 8–12 h overnight fast. The pre-analytical, analytical and post analytical factors that interfere with the results of the biochemical measurements were controlled and maintained by a medical laboratory technologist performing the laboratory analysis. Moreover, commercially prepared quality control samples were used daily prior to running the study samples in order to assess the function of the instrument’s, laboratory chemicals, and procedural performances with strict adherence to standard operating procedures. The blood pressure status was measured using a standardized and calibrated sphygmomanometer with appropriate cuff sizes. The apparatus was calibrated daily before data collection.

### Data analysis and presentation

The data were cleaned and then entered into Epi Data 3.1, and then exported to the Statistical Package for Social Sciences (SPSS), Version 21 for statistical analysis. The Chi-square test and Student’s t-test were used respectively to compare categorical and continuous variables between the two study groups. Logistic regression analysis was used to analyze factors associated with metabolic syndrome. A bivariable analysis with a p-value < 0.25 and variables considered medically significant were candidates for multivariable logistic regression analysis. Odds ratios and 95% confidence intervals (CIs) were calculated to determine the independent risk factors associated with MetS. Data were presented using narrative, figures, and tables from the results of the statistical analysis. Statistical significance was set at p < 0.05.

## Results

### Socio—demographic characteristics of the study groups

A total of 192 study participants (96 psychiatric and 96 non-psychiatric individuals) were interviewed. The mean age (years) of psychiatric patients was 37.18 ± 12.59 whereas, 36.59 ± 13.56 for non-psychiatric individuals, which was a statistically insignificant difference between the two groups (p = 0.754). Around 32.2% of psychiatric patients and 44.8% of non-psychiatric study participants attended college and above the educational level. The majority of psychiatric study participants were unmarried (51.1%) and rural dwellers (61.5%). Moreover, the vast majority of exposed study participants (75%) were unemployed as compared to their non-exposed counterparts (37.5%) (Table 1).

**Table 1:** Socio- demographic characteristics of the study groups, Eastern Ethiopia, 2021

Characteristics	Psychiatric patients Number = 96 Number (%)	Non-psychiatric patients Number = 96 Number (%)	*p* value
Sex			0.561**
Male	55 (57.3%)	56 (58.3%)	
Female	41 (42.7%)	40 (41.7%)	
Age (mean ± SD)	37.18 ± 12.59	36.59 ± 13.56	0.754**
Educational status			0.076*
No formal education	45 (46.9%)	26 (27.1%)	
Primary education	3 (3.1%)	6 (6.3%)	
Secondary education	17 (17.7%)	21 (21.9%)	
College & above	31 (32.2%)	43 (44.8%)	
Marital status			0.743*
Single/unmarried	35 (36.5%)	33 (34.4%)	
Married	12 (12.5%)	33 (34.4%)	
Divorced/separated/died	12 (12.5%)	13 (13.5%)	
Residence			0.009*
Rural	59 (61.5%)	41 (42.7%)	
Urban	37 (38.5%)	55 (57.3%)	
Employment			0.061*
Unemployed	72 (75.0%)	36 (37.5%)	
Employed	24 (25.0%)	60 (62.5%)	
Ever smoked			0.86*
No	79 (82.3%)	82 (85.4%)	
Yes	17 (17.7%)		
Ever Alcohol Intake			0.401*
No	70 (72.9%)	75 (78.1%)	
Yes	26 (27.1%)	21 (21.9%)	
Regular physical activity			0.521*
Yes	39 (40.6%)	29 (30.2%)	
No	57 (59.4%)	67 (69.8%)	
Family history of hypertension			0.082*
Yes	36 (37.5%)	13 (13.5%)	
No	69 (71.9%)	83 (86.5%)	
Family history of diabetes			0.013*
Yes	27 (28.1%)	13 (13.5%)	
No	68 (70.8%)	83 (86.5%)	

*SD * Standard deviation

**Independent sample T test, *Pearson’s chi-square test

### The magnitude of metabolic syndrome

The magnitude of metabolic syndrome among psychiatric exposed patients was 36.5%, whereas it was 21.9% among non-exposed controls (p = 0.0026). In addition, the components of metabolic disorders among psychiatric exposed patients were more likely to have a broader waist circumference, reduced HDL-C level, higher systolic blood pressure, and higher fasting blood glucose levels compared to the non-psychiatric control clients, whose corresponding burdens were: 25.0% vs. 14.3% (p = 0.01), 35.4% vs. 20.8% (p = 017), 41.7% vs. 29.2% (p = 0.022), 40.6% vs. However, serum triglycerides and diastolic pressure showed statistically insignificant differences between psychiatric and non-psychiatric patients (Table 2).

**Table 2:** Metabolic disorder and its components among the study groups, Eastern Ethiopia, 2021

Characteristics	Psychiatric patients (exposed)	95%CI	Non-psychiatric	95% CI	P value
Overall magnitude **MetS**	35 (36.5%)	(27.6, 47.4)	21 (21.9%)	(13.5, 30.3)	0.026
Increased waist circumference (males > 102 cm, females > 88 cm)	24 (25.0%)	(17.2, 35.4)	10 (14.3%)	(7.8, 23.0)	0.010
High systolic blood pressure (≥ 130 mm Hg)	40 (41.7%)	(17.2, 31.7)	28 (29.2%)	(22.4, 38.5)	0.022
High diastolic blood pressure (≥ 85 mmHg)	28 (29.2%)	(20.8, 39.1)	18 (18.8%)	(13.5, 27.6)	0.091
Raised triglycerides (> 150 mg/dl)	37 (38.5%)	(29.2, 48.5)	26 (27.1%)	(19.8, 39.1)	0.270
High fasting plasma glucose (≥ 110 mg/dl)	39 (40.6%)	(21.7, 40.1)	18 (18.8%)	(10.4, 25.0)	0.001
Low HDL levels (males < 40 mg/dl, females < 50 mg/dl)	34 (35.4%)	(24.0, 42.8)	20 (20.8%)	(19.2, 34.4)	0.017

Statically significant (p < 0.05), *HDL* high density lipoprotein, *MetS* Metabolic syndrome

### Predictors of metabolic syndrome among psychiatric patients

All variables were entered into a binary logistic regression to select candidate variables. All covariates with a p value of less than 0.25 in the bivariate analysis were included in the multivariable analysis. Hence, bivariate analysis revealed that being female, age of study participants, place of residence, educational status, alcohol consumption, physical inactivity, duration of psychiatric illness, and family history of hypertension were associated with metabolic syndrome at p value < 0.25. Multivariable analysis showed that age greater than 50 years (AOR: 2.8, CI: 1.14, 20.0, p < 0.05); being female study participants (AOR: 7.4, CI: 2.0, 27.6, p < 0.05), being urban residence (AOR: 6.4, CI: 2.2, 20.6, p < 0.05), alcohol intake (AOR: 5.3, CI: 1.3, 21.2), being physically inactive (AOR: 3.8, CI: 1.1, 12.9, p < 0.05) and family history of hypertension (AOR: 3.8, CI:1.1, 12.2, p < 0.05)were independent predictors of MetS among psychiatric patients (p < 0.05) (Table 3).

**Table 3:** Factors associated with metabolic syndrome among psychiatric patients at Dilchora Hospital psychiatric center, Eastern Ethiopia, 2021

Parameter	Category	MetS		COR (95% CI)	AOR (95%CI)
		Yes	No		
Age (years)	18–30	6 (20.0%)	24 (80%) 1	1	1
	31–40	7 (26.9%)	9 (73.1%)	0.97 (0.6–1.5)	0.33 (0.06, 2.28)
	41–50	10 (47.6%)	11 (52.4%)	0.57 (0.3–0.89)	1.1 (0.18, 7.24)
	> 50	12 (63.2%)	7 (36.8%)	6.8 (1.88–24.9)	2.8 (1.04, 20.0)*
Gender	Male	13 (25%)	39 (75.0%)	1	1
	Female	22 (50.0%)	22 (50.0%)	3 (1.26, 17.10)	7.4 (2.0, 27.6)*
Education	Uneducated	23 (51.1%)	22 (48.9%)	3 (1.11, 8.1)	5.3 (0.1.0, 25.5)
	Primary	1 (33.3%)	2 (66.7%)	1.4 (0.11, 18.07)	4.7 (0.11, 18)
	Secondary	3 (17.6%)	66 (0.14, 2.7)	14 (82.4%)	2.3 (0.31, 17.3)
	College and above	8 (25.8%)	23 (74.2%)	1	1
Residency	Rural	11 (18.6%)	48 (81.4%)	1	1
	Urban	24 (64.9%)	13 (35.1%)	8 (3.14, 20.6)	6.4 (2.2, 20.6)*
Ever smoked	No	26 (32.9%)	53 (67.1%)	1	1
	Yes	9 (52.9%)	8 (47.1%)	2.29 (0.79, 6.6)	2.3 (0.36, 17.8)
Ever taken alcohol	No	19 (27.1%)	51 (72.9%)	1	1
	Yes	16 (61.5%)	10 (38.5%)	4.29 (1.66, 11.10)	5.3 (1.3, 21.2)*
Regular physical activity	Yes	8 (20.5%)	31 (79.5%)	1	1
	No	27 (47.4%)	30 (52.6%)	3.48 (1.36, 8.88)	3.8 (1.1, 12.9)*
Family history of hypertension	Yes	18 (50.0%)	18 (50.0%)	2.52 (1.06, 5.98)	3.8 (1.1, 12.2)*
	No	17 (28.3%)	43 (71.7%)	1	1
Duration of mental illness (years)	< 5	6 (24.0%)	19 (76.0%)	1	1
	5–10	5 (22.7%)	17 (77.3%)	0.931 (0.24, 3.612)	0.45 (0.04, 4.2)
	> 10	24 (49.0%)	25 (51.0%)	3.04 (1.03, 8.9)	0.58 (0.10, 3.18)

*MetS* metabolic syndrome, *COR* crude odds ratio, *AOR* adjusted odds ratio

* = p < 0.05

## Discussion

Ethiopia is a developing sub-Saharan country; like other developing countries, it is going through an epidemiological transition from communicable to non-communicable diseases in general. This comparative cross-sectional study identified a high prevalence of metabolic syndrome among severely ill psychiatric patients compared to non-psychiatric individuals, which was 35.6% and 21.9%, respectively. This finding was consistent with the studies reported in Qatar [26], South Africa [27], and Egypt [28] which found a higher magnitude of metabolic syndrome among psychiatric-exposed patients compared with non-psychiatric study participants. High burden of metabolic syndrome among psychiatric clients could be due to psychiatric patients having less physical activity due to functional disorders, psychological stress, excessive alcohol intake, excessive smoking, and inadequate medical care have been implicated [7–9].

The overall prevalence of metabolic syndrome among psychiatric patients in the current study was (35.6%) comparable with different studies: 28.9% at Jimma University Medical Center, Ethiopia [21], 34.74% in Kashmir [29] and 43.6% in Palestine [30]. However, the prevalence estimated in the present study was much higher than in studies conducted in Hawassa, 24.5% [31], 23.51% in Uganda [15], and 24.3% in Brazil [32]. The possible difference could be due to differences in types or generation of antipsychotic agents used by the patients (in our study, most of the patients used first-generation antipsychotics at the time of study), period of study, patients’ lifestyle, patterns of psychiatric illness (in our study, patients with severe psychiatric disorder were considered), duration of psychiatric illness, and sociocultural differences [33–35].

In our study, most of the metabolic syndrome components were highly frequent in patients with psychiatric disorders compared to non-psychiatric controls. The magnitude of waist circumference was significantly higher in patients with severe psychiatric illness than in non-psychiatric controls, which was 25% vs. 14.3%, p = 0.03. The magnitude of fasting blood glucose was significantly higher among patients with severe psychiatric disorders (40.6%) compared to the non-psychiatric controls (18.8%), p = 0.001. The magnitude of reduced high density lipoprotein dyslipidemia was significantly higher among severely ill psychiatric patients (35.4%) than among non-psychiatric controls (20.8%). In addition, the magnitude of systolic blood pressure was higher in severely ill psychiatric patients (41.7%) than in non-psychiatric controls (29.2%), p = 0.022. This was comparable to the study conducted in Egypt in 2017; almost all components of metabolic syndrome were significantly higher in psychiatric patients than in non-psychiatric individuals [26, 36–42].

Higher odds of having metabolic syndrome were found in female subjects, the advanced age group, subjects who were urban residents, physically inactive individuals, and subjects who were alcohol consumers. Participants over the age of 50 were found to be significantly more likely to have MetS (AOR: 2.8, CI: 1.14, 20.0, P 0.05). Muscle, fat, and other tissues may become less sensitive to insulin as we age, resulting in dysglycemia and dyslipidemia [43]. And also, as age increases, arteries get stiffer, so blood pressure goes up due to loss of elastin fibers and accumulation of stiffer collagen fibers in the media of large arteries [44]. This finding is similar to a previous study reported in Kashmir [29] and Jimma, Ethiopia [21]. In contrast, a study conducted in Ghana discovered that age (> 53 years) was statistically insignificant in the development of metabolic syndrome [45].We speculate that it could be due to variations in socioeconomic factors and health seeking behaviors among the two study populations.

In addition, the present study revealed that participants of female sex were at higher risk of developing metabolic syndrome than those of male participants (AOR: 7.4, CI: 2.0, 27.6, p < 0.05). This result is consistent with the studies conducted in Kashmir [29], South Africa [46], South West Ethiopis [21] and Southern-Ethiopia [31]. Possible explanations for this gender disparity include differences in physiology, level of physical activity, and psychological factors between the male and female sexes.

Urban residency was found to be an important risk factor for metabolic syndrome (AOR: 6.4, CI: 2.2, 20.6, p < 0.05). This could be explained by the fact that urban residency associated with physical inactivity and consumption of calorie-rich diets contributed to the pathogenesis of metabolic syndrome. This finding is consistent with a study conducted in Poland [47] and Bangladesh [48].

The present study also showed a significant relationship between alcohol consumption and the development of MetS (AOR: 5.3, CI: 1.3, 21.2). The possible reason might be associated with excessive caloric intake through alcoholic consumption leads to adipocyte hypertrophy and increases visceral adipose tissue. Adipose tissue possibly secretes many cytokines and reactive oxygen radicals, thus promoting inflammation and insulin resistance syndrome [44, 49, 50]. This finding was in line with the studies conducted in Southern Ethiopia [31] and Venezuela [51] ever alcohol intake was significantly associated with metabolic syndrome. However, this finding was in contrast with the study conducted in Jimma that found alcohol intake was not associated with metabolic syndrome [21].We speculate the difference could be due to the time variation, the socio-economic and cultural factors observed between the study participants.

In the present study, participants with low physical activity were at a higher risk of developing metabolic syndrome (AOR: 3.52, CI: 1.1, 12.9, p < 0.05). This finding was comparable with similar studies in Taiwan in 2016 [52] and China [53]. Conversely, there was an inconsistent report from Uganda in 2017 that said the level of physical activity was not associated with the development of metabolic syndrome [26].The possible justification could be partly due to information relying on self-reporting data, which is prone to information bias, sociodemographic variation among the two study groups. The current study also showed that the risk of developing MetS was 2.5 times greater than having a family history of hypertension. This finding is in line with the reports of previous studies in Sri Lanka in 2015 [54] and Ghana in 2018 [55]. The possible reason might be associated with blood relatives, who tend to have many of the same genes that can predispose a person to MetS, or relatives may also share some of the same habits such as diet, exercise, and substance use that can affect risk [56].

## Strengths and limitations of the study

As per the knowledge of the researchers, this study is one of the first analytical comparative studies to assess the magnitude of metabolic disorder and its associated factors among patients with severe psychiatric disorder and non-psychiatric controls in Ethiopia. As a result, the project investigated the prevalence of metabolic disorders in patients with psychiatric disorders, who are less frequently studied for these disorders than age- and gender-matched non-psychiatric patients who are thought to be representative of similar settings. We compared our results with those of age-and sex-matched "non-psychiatric" controls. This study investigated several variables, which can also influence the burden of metabolic disorders in patients with a psychiatric disorder. In addition, it was assured that biochemical analysis was done in clinical chemistry laboratory machines that passed the laboratory quality management system and were accredited by the Ethiopian National Accreditation Office by following the requirements of ISO: 2020 for technical competency. Our data was supplemented by clinical measurements with biochemical analysis that might raise the reliability/accuracy of the estimate or diagnosis of metabolic disorder.

Our study had some limitations. It was conducted in one health facility, and findings from the study may not be generalizable beyond the population of patients attending Dilchora referral hospital. The study may have been subjected to social desirability/acceptability bias from study participants wanting to provide socially desirable responses to questions about risky lifestyles such as smoking and alcohol consumption. We did not assess the dietary habits of the study participants, yet these are important determinants of metabolic disorders.

## Conclusions

In Dilchora Hospital, Eastern Ethiopia, metabolic syndrome and its components are highly prevalent among psychiatric patients. Early detection and appropriate intervention are mandatory among patients with advanced age, female sex, urban residency, alcohol consumers, physically inactive individuals, and family history of hypertension. Frequent clinical and biochemical monitoring should, therefore, form part of care in patients with psychiatric disorders on follow-up visits. In addition, routine assessment of sociodemographic, behavioral, and biological risk factors should be institutionalized to address the burden of metabolic syndrome. Furthermore, future prospective research should be implemented to identify the relationship between psychiatric illnesses and metabolic syndrome.

## Data Availability

The data used to support the findings of this research are available on request from the corresponding author.

## Abbreviations

BMI: Body mass index
DCRH: Dilchora Referral Hospital
DBP: Diastolic blood pressure
MetS: Metabolic syndrome
HDL-c: High Density Lipoprotein cholesterol
NCEP/ATP3: National Cholesterol Education Adult Treatment Panel III
SBP: Systolic blood pressure
